# Antimicrobial Resistance: Is There a ‘Light’ at the End of the Tunnel?

**DOI:** 10.3390/antibiotics12091437

**Published:** 2023-09-12

**Authors:** Leon G. Leanse, Sanjay Marasini, Carolina dos Anjos, Tianhong Dai

**Affiliations:** 1 Health and Sports Sciences Hub, University of Gibraltar, Europa Point Campus, Gibraltar GX11 1AA, Gibraltar; 2 Wellman Center for Photomedicine, Massachusetts General Hospital, Harvard Medical School, Boston, MA 02114, USA; cdosanjos@mgh.harvard.edu (C.d.A.); tdai@mgh.harvard.edu (T.D.); 3 New Zealand National Eye Centre, Department of Ophthalmology, The University of Auckland, Auckland 1142, New Zealand; s.marasini@auckland.ac.nz

**Keywords:** antimicrobial resistance, intrinsic resistance, phenotypic resistance, acquired resistance, phototherapy, photodynamic therapy, antimicrobial blue light, ultraviolet light

## Abstract

In recent years, with the increases in microorganisms that express a multitude of antimicrobial resistance (AMR) mechanisms, the threat of antimicrobial resistance in the global population has reached critical levels. The introduction of the COVID-19 pandemic has further contributed to the influx of infections caused by multidrug-resistant organisms (MDROs), which has placed significant pressure on healthcare systems. For over a century, the potential for light-based approaches targeted at combatting both cancer and infectious diseases has been proposed. They offer effective killing of microbial pathogens, regardless of AMR status, and have not typically been associated with high propensities of resistance development. To that end, the goal of this review is to describe the different mechanisms that drive AMR, including intrinsic, phenotypic, and acquired resistance mechanisms. Additionally, the different light-based approaches, including antimicrobial photodynamic therapy (aPDT), antimicrobial blue light (aBL), and ultraviolet (UV) light, will be discussed as potential alternatives or adjunct therapies with conventional antimicrobials. Lastly, we will evaluate the feasibility and requirements associated with integration of light-based approaches into the clinical pipeline.

Due to antimicrobial resistance (AMR) the treatment of infectious diseases has compromised the safety of public health [1] and the COVID-19 pandemic has made the consequences of AMR more prominent [2]. While complexities remain with the timely diagnosis and management of multidrug-resistant organisms (MDROs), it is essential that strategies to expedite and refine the diagnostic and treatment paradigms of infectious diseases are explored [1]. This review aims to highlight, in detail, antimicrobial resistance, discuss its mechanisms, and consider health impacts. The aim is also to discuss light-based anti-infective agents as innovative and potentially effective antimicrobial strategies to manage MDROs. Lastly, we will discuss our perspective on the future of light-based technology in the fight against AMR.

## 1. Antimicrobial Resistance: How Do Microbes Evade Antimicrobial Agents?

Typically, when we consider an organism to be ‘sensitive’ to an antimicrobial therapeutic, it is defined clinically by two specific determinations, the minimum inhibitory concentration (MIC) and minimum bactericidal concentration (MBC) [3]. The MIC is defined as the lowest concentration of antimicrobial therapy required to visibly inhibit the growth of microorganisms (e.g., bacteria). Clinically, MBC is less commonly used. It is defined as the lowest antimicrobial concentration that can reduce the viability of a microorganism by at least 3-log_10_ colony-forming units/mL (CFU/mL) or by 99.9%.

Antimicrobial resistance is a process by which different agents of infection, bacteria, viruses, fungi, and protozoans resist their conventional antimicrobial treatment [1]. More appropriately, it occurs when the eradication of an infectious organism can only be achieved at concentrations of a therapeutic agent significantly higher than what may be applied safely [4]. Microorganisms that harbor resistance mechanisms to at least one agent within at least three antibiotics classes are often classed as multidrug-resistant (MDR) organisms [5]. For a bacterial pathogen, the potential for administration of antibiotics would be determined by a defined MIC that would be used to predict whether a particular antibiotic may work effectively [4]. Taking *Staphylococcus aureus* as an example, common last resort antibiotic would be Vancomycin, a glycopeptide antibiotic that binds to the d-Ala-d-Ala dipeptide of the peptidoglycan (PG)-stem unit [6]. For example, the typical characteristics for susceptibility to Vancomycin would be an MIC of 1 μg/mL. Increasing the MIC to 4–8 μg/mL would make vancomycin intermediate *S. aureus* (VISA) and exceeding 16 μg/mL would be classed as vancomycin resistant *S. aureus* (VRSA) [6].

Resistance to antibiotics/antimicrobials can occur either as intrinsic, phenotypic, or acquired. In this section, we will summarize different mechanisms that underpin antimicrobial resistance.

### 1.1. Intrinsic Resistance

An antimicrobial application typically relies on the attachment to a specific target to interrupt its function [7]. For example, penicillin antibiotics that belong to the wider β-lactam class of antibiotics interrupt peptidoglycan synthesis by binding to penicillin-binding protein (PBP), an essential constituent of cell wall biogenesis [8]. Different microorganisms, however, contain structural, biochemical, physiological, or metabolic differences that may not permit the functionality of a particular antimicrobial due to an inability to bind to their specific targets [9]. These differences may be due to the absence of that specific target (the cell wall in yeasts, for example, are composed of chitin [10] and not peptidoglycan, rendering penicillin ineffective in their treatment), or they may possess a target that is out of reach of a specific antibiotic (e.g., a selectively impermeable outer membrane that blocks the entrance of certain antimicrobials) [11].

Bacteria are conventionally characterized as ‘Gram-positive’ or ‘Gram-negative’ [12]. This characterization was from a present ‘Gold-standard’ bacterial staining technique invented by Hans Christian Gram in 1882, who used crystal violet coupled with iodine and a second dye (safranin or fuchsine), coupled with various ‘de-staining’ techniques, to identify different bacterial morphologies [12]. The principle was that the peptidoglycan present within the cell wall of bacteria would visibly retain crystal violet stain. As Gram-positive bacteria possess a greater quantity of peptidoglycan, they look purple under the microscope. Gram-negative bacteria, however, retain the second dye (safranin/fuchsine), which is responsible for the characteristic pink color [12].

Unlike their Gram-positive counterparts, Gram-negative bacteria possess an extra membrane and lipopolysaccharide that make its outermost layer, exterior to a thick periplasmic space and a thin peptidoglycan layer [11]. This outer membrane is relatively impermeable and limits the entrance of certain antibiotics, which reduces their ability to bind to specific targets (e.g., peptidoglycan). Therefore, Gram-negative bacteria, for example, would be ‘intrinsically’ resistant to certain antibiotics (such as glycopeptides) due to the reduced ability of antibiotics to effectively bypass the outer membrane [13].

This concept of intrinsic antimicrobial resistance is especially important when selecting the appropriate antimicrobial treatment, thus emphasizing the importance of obtaining an accurate diagnosis [14].

### 1.2. Phenotypic Strategies That Induce Antimicrobial Resistance

All organisms have evolved to resist various environmental stressors or conditions to promote survival [15]. Bacteria, for example, have developed highly specified responses stimulated by changes in environmental conditions. When bacteria colonize a host to achieve an infection, their replication induces the expression of a variety of autoinducer molecules, which, when reach a critical mass, can induce the production of virulence factors, such as biofilms [16]. This process is known as quorum sensing, a form of intra- or interspecies communication that allows bacteria to propagate their line.

Bacterial biofilms are communities of bacteria that are encased within an extracellular polymeric substance (EPS) [17]. As mentioned above, they are produced as a survival mechanism, either aiding in establishing infection or permitting survival on abiotic surfaces to aid their transmission [17]. Biofilms are particularly complex in that they comprise both the EPS and metabolically dormant ‘core’ that effectively evades both the immune system (via sequestration) and antibiotic attack through a reduced metabolism (which limits antibiotic efficacy) and the structural EPS impediment, which limits immune cell and antibiotic infiltration [18].

Biofilms are not only virulence factors aiding in infection establishment or transmission but also important mechanisms for phenotypic resistance, posing a significant concern as they are often implicated as one of the drivers of infection recurrence or recalcitrance [19]. Because they can be up to 1000× more resistant to antibiotics than their planktonic or ‘free-floating’ counterparts [20], the host may retain sub-populations of bacteria even after treatment, permitting the re-establishment of infection.

Bacterial persister cells are another example of phenotypic resistance found in bacteria and as their name suggests, they are the principal cause of persistent infections [21]. When bacteria are stressed by nutrient limitation or excessive antibiotics, they undergo a process that renders them ‘dormant’, shutting down their metabolic processes [21]. This process serves multiple functions, including limiting the nutrients supply required to survive during nutrient restriction. Another important consequence of persister cell formation is tolerance to antibiotics and other chemical stressors that threaten survival. Like biofilms, persister cells are up to 1000× more tolerant to antibiotic attack than their metabolically active counterparts [22].

Antibiotics work by disrupting metabolic activity and replication and it is an intelligent evolutionary response of bacteria to restrict metabolic activity to limit antibiotic effectiveness and ensure their survival [22]. As with biofilms, the presence of persister cells within the subpopulation of bacteria permits infection recurrence due to the inability of antibiotics to eradicate the entire population [21].

### 1.3. Acquired Resistance

When we consider AMR as a threat to public health, it is typically acquired resistance that is being referred to, as opposed to ‘intrinsic’ or ‘phenotypic’ mechanisms described above. Acquired resistance is a process by which a previously sensitive microorganism develops resistance (encoded genetically) to a conventionally used antimicrobial agent [23]. This process of acquired resistance can occur over time because of over-exposure to sublethal antimicrobial concentrations or horizontal gene transfer (on plasmids and integrative conjugative elements) when specific resistance mechanisms are transferred between microbes [23].

#### 1.3.1. Single Nucleotide Polymorphisms Drives of Resistance through Selective Pressure

Due to the high rate of microbial replication, with bacteria being especially rapid in their propagation, their propensity to developing mutations is equally high [24]. Mutations beneficial to bacterial survival are often retained, while others with no positive effects are not specifically selected. For example, when bacteria are in the presence of certain antibiotics, over time, they develop ‘base’ changes or single nucleotide polymorphisms ‘SNPs’ that alter the structure of their encoded protein to prevent or limit the ability of an antibiotic to bind to the select target [25]. During antibiotic exposure, SNPs occur stochastically; however, when these SNPs develop at the specific antibiotic targets (e.g., penicillin binding protein), selective pressure ensures their retention [26]. As the SNPs allow the microorganism to survive, they can propagate within the population and retain resistance to a particular antibiotic by which they were over-exposed [27].

#### 1.3.2. Expression of Enzymes That Modify or Hydrolyze Antibiotics

The ability of a bacterium to express enzymes that either hydrolyze or modify antibiotics to negate their functionality remains a clinically important resistance mechanism [28]. Although β-lactam antibiotics remain at the forefront of our antimicrobial portfolio, perhaps the most important enzyme-mediated antimicrobial resistance mechanism occurs through β-lactamase expression [29]. Various classes of β-lactamases encoded on plasmids or integrative conjugative elements, disseminated across numerous pathogenic bacterial species, are particularly important to Gram-negative bacteria [30]. Within these bacteria, β-lactamases functionally inactivate β-lactam antibiotics via hydrolysis of amide bonds of their β-lactam rings. β-lactamases may be divided into four specific Ambler classes, namely, those that are divided into active-site serine β-lactamases, which comprise three Ambler classes (A, C, and D), and Metallo- β-lactamases (also referred to as ‘zinc-dependent’), which belong to Ambler class B [30].

As with enzymes that can hydrolyze antibiotics to negate their function, some can modify certain antibiotic targets by adding specific chemical moieties that prevent the binding of antibiotics to their molecular targets via steric hindrance [31]. The antibiotics that are most affected by enzyme modification are those that elicit their function via ribosome binding [28]. The principal enzymatic mechanisms of modification are acetylation, which affects chloramphenicol, streptogramins, and aminoglycosides; phosphorylation, which impacts chloramphenicol and aminoglycosides; and adenylation, which affects lincosamides and aminoglycosides [32]. For example, aminoglycoside-modifying enzymes (AMEs) modify aminoglycosides via changes in amino/hydroxyl groups present within the molecule. AMEs represent a dominant mechanism of resistance that can be found globally and are often contained within mobile genetic elements, although they can also be retained within the chromosomes of certain bacterial species, such as *Enterococcus faecium* [32].

#### 1.3.3. Efflux Pump Expression to Eject Antibiotics

Arguably, one of the most important resistance mechanisms is the expression of efflux pumps [33]. Efflux pumps are sophisticated machinery expressed by bacteria that can eject toxic compounds or antibiotics out of the cell [33]. They are found in both Gram-negative and Gram-positive bacteria and can either be highly specific, such as the *tet* system [34], responding solely to the presence of tetracycline, or have a broad spectrum of efflux capability, which are often expressed by multidrug-resistant bacteria [35].

**Figure 1:**
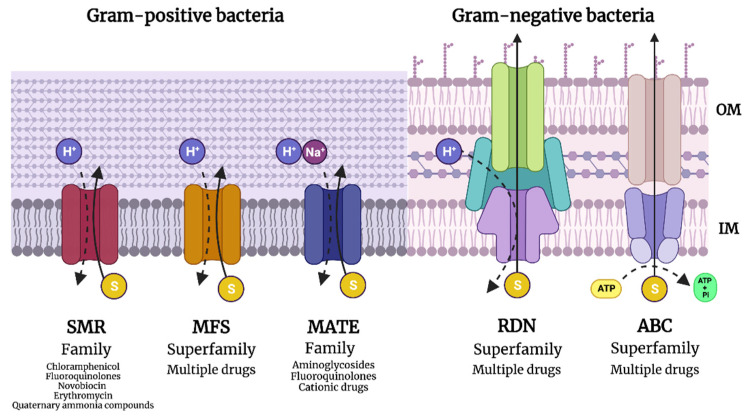
Diagram showing the different families of efflux pumps. Created on BioRender.com.

Efflux pumps may be divided into five dominant families, including the small multidrug-resistance family (SMR), the major facilitator superfamily (MFS), the resistance–nodulation–cell division superfamily (RND), the ATP-binding cassette family, and the multidrug and toxic compound family (Figure 1). Each branch of the efflux pumps varies structurally and physiologically to conform to their specific requirements for the ejection of their substrates [36].

The most well-characterized pump that drives efflux-mediated resistance is the *tet* system [34]. It is part of the major facilitator superfamily, which becomes expressed only in the presence of tetracycline (via *tetR* regulation). It extrudes tetracycline via a process of proton exchange which fuels the energy requirements. Over 20 tet genes have been characterized, predominantly carried on plasmids, although they can also be found chromosomally, potentially being carried on integrative conjugative elements [37]. Most of these are found within Gram-negative bacteria, with *tetK* and *tetL* being dominant within Gram-positive bacteria. Interestingly, these pumps can eject tetracycline and doxycycline but do not affect tigecycline or minocycline, thus not impacting their relative antimicrobial susceptibilities. There are also more generalized multidrug-resistance efflux pumps [36] expressed by numerous bacteria that can extrude many different antibiotics. Examples include the AcrAB-TolC (found within the *Enterobacteriaceae*) [38], MexAB-OprM (harbored by *P. aeruginosa*) [39], etc.

## 2. Light-Based Anti-Infective Agents: Can They Overcome Antimicrobial Resistance?

During the late 19th century, Niels Ryberg Finsen postulated the therapeutic potential of light [40]. He was suffering from Pick’s disease, which is characterized by the thickening of connective tissues in spleen, heart, and liver, that eventually results in loss of function. He showed interest in using light as a therapy to his debilitating disease as his symptoms were relieved after spending more time in the sun. He investigated the use of red light to treat smallpox and Lupus vulgaris [41]. His works on ‘phototherapy’ eventually won him the Nobel Prize in Physiology and Medicine in 1903 [41].

Since Finsen discovered the therapeutic effects of light, there have been multiple studies that used light to treat diseases, including infectious diseases. Light has been studied in combination with chemical ‘photosensitizers’ to generate reactive oxygen species that can eradicate pathogens [42]. Additionally, the intrinsic antimicrobial effects of blue light [43] and ultraviolet light [44] have been explored as strategies to kill microorganisms [44]. Furthermore, light has been explored in combination with other pharmacological agents, such as antibiotics, to potentiate their effects [43]. To that end, this section aims to summarize the advances made in photodynamic therapy, antimicrobial blue light, and ultraviolet radiation as strategies to overcome antimicrobial resistance.

### 2.1. Antimicrobial Photodynamic Therapy

**Figure 2:**
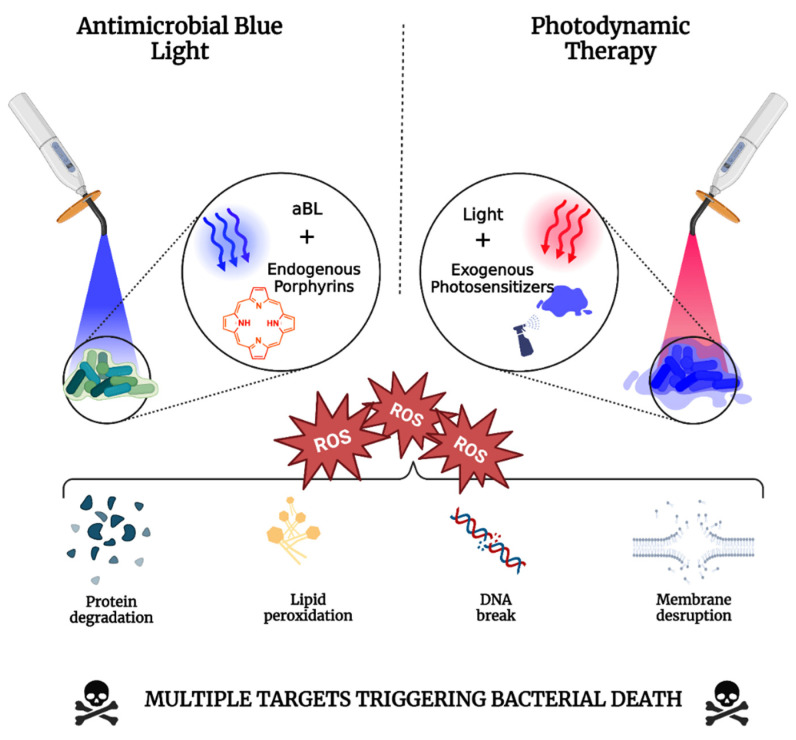
Diagram illustrating the mechanisms of aBL and PDT against bacteria. Created on BioRender.com.

Photodynamic therapy (PDT) is defined as a photochemical process that combines light with a chemical photosensitizer (PS) in the presence of oxygen to inactivate or destroy cells (e.g., cancer cells and microbial cells) (Figure 2) [45]. This phenomenon was first discovered by a medical student in Munich called Oscar Raab, who investigated the effects of acridine red on the protist *Paramecium* spp. [46]. He found that when using small concentrations of the dye, the toxicity towards the protist was highly inconsistent despite numerous replicates. It was later elucidated by Herman von Tappeiner, his supervisor at the time, that these inconsistencies were due to daylight changes. In 1905 after realizing this phenomenon, Herman Von Tappeiner, in collaboration with Albert Jesionek, applied acridine red in combination with sunlight to treat skin carcinomas, later coining the term ‘photodynamic phenomenon’ [47]. The same year, von Tappeiner applied this technique against bacteria, being the first person to demonstrate bacterial inactivation using PDT approach. Over the past several decades, many studies have used aPDT to treat localized infections [48,49,50]. Literature suggests that bacteria do not develop resistance to aPDT as they do with antibiotics [51], and aPDT appears to eliminate pathogens that are resistant to conventional antibiotics [52]. It potentially makes aPDT an attractive approach to combat infectious diseases, particularly in ‘post-antibiotic era’. It is important to note, however, that in recent years, increasing evidence has shown that aPDT may induce certain ‘tolerance’ mechanisms that might hinder its application if used inappropriately [53]. To that end, in this section, we will summarize the mechanisms, commonly applied light wavelengths and photosensitizers, the resistance potential of microbes, and the safety of PDT for eliminating pathogens.

#### 2.1.1. Mechanisms of Photodynamic Therapy

Classically, the photodynamic processes are split into two photochemical pathways, denoted by the ‘Type I’ and/or ‘Type II’ reaction [54] (Figure 1). The Type I reaction typically begins with the ground state PS, which, when excited by light, results in electron transfer that induces the production of superoxide (O_2_^−^). Production of O_2_^−^, in itself, is not thought to be primarily responsible for oxidative damage elicited via a typical photodynamic reaction. Rather, the O_2_^−^ anion can self-react to generate hydrogen peroxide (H_2_O_2_) and oxygen via a dismutation process typically catalyzed by superoxide dismutase. This principal step occurs before the highly microbicidal ^•^OH radicals are generated, which require further O_2_^−^ to function as the reducing agent. During this step, O_2_^−^ reduces ferric iron (Fe^3+^) via electron donation, which can then catalyze the Fenton reaction that converts H_2_O_2_ to ^•^OH radicals.

Additionally, O_2_^−^ can potentially react with the generated ^•^OH radicals to generate a variety of other reactive oxygen species or free radicals, such as singlet oxygen (^1^O_2_), nitric oxide, or peroxynitrite, all of which induce significant oxidative stress [55]. In the ‘Type II’ reaction, which often occurs in tandem with the ‘Type I’ mechanism, the production of ^1^O_2_ is the most prominent ROS generated. When ^•^OH diffuses through cells, it can be added to a substrate that contains carbon (such as a fatty acid), which forms another radical defined as a hydroxylated adduct. ^•^OH can also oxidize ground-state molecular oxygen, generating the peroxyl radical (ROO), which can damage numerous substrates such as fatty acids/lipids. These ROS (in conjunction with ^1^O_2_) are generated via the Type II pathway and can result in myriad reactions that impact molecules, including proteins, lipids, nucleic acids, etc., present within cells/bacteria, leading to viability losses [55].

Previously, the Type I and Type II pathways of PDT were thought to be the only mechanisms that could drive the photodynamic process [56]. It has been suggested that these pathways may suffer from a drawback in that they are ‘oxygen dependent’ and may be limited in their therapeutic application towards infections within hypoxic or anaerobic environments. Therefore, in recent years, work into developing or exploiting a novel Type III pathway that is oxygen independent for microbial inactivation has been proposed to overcome low oxygen saturation that surrounds anaerobic infections (such as those caused by obligate anaerobes, e.g., *Porphyromonas gingivalis*, *Clostridium* spp., etc.) [56]. Several chemical photosensitizers/PDT enhancers have been described in the literature (e.g., tetracyclines, inorganic salts, etc.) that may follow this novel Type III photochemistry. Psoralens, for example, belong to the group of compounds called furanocoumarins that are frequently found in plants [57]. Psoralen was previously applied with Ultraviolet A (UVA) to treat various skin diseases. However, this practice ceased with expanding knowledge demonstrating the link between UVA and skin cancer [56]. Mechanistically, however, this is interesting because although both photons and drugs are applied, it diverges from the conventional photodynamic process. Psoralens bind to DNA, and upon irradiation with UVA, it becomes activated and modified to produce 3,4- or 4,5-mono-adducts, which can absorb UVA, which is a completely oxygen-independent process. [55]. It was found that when 8-methoxy psorelean was combined with UVA against bacteria (*S. aureus* and *E. coli*), the efficacy of inactivation could be enhanced 10-fold when oxygen was removed and replaced with nitrogen [58]. Their comparator studies with methylene blue plus red light observed the opposite, with a severe reduction in efficacy (approximately reduced 10,000-fold) when oxygen was removed and replaced with nitrogen. These findings demonstrated that unlike methylene blue, which follows the typical Type I/Type II pathways of photodynamic therapy, psoralen plus UVA appears to not only not require oxygen but is also more efficacious in its absence.

#### 2.1.2. Photodynamic Inactivation of Microbes: A Function of Wavelength and Photosensitizer

Since antimicrobial photodynamic therapy (aPDT) was first applied over a century ago, there have been ever-expanding number of studies in the literature that demonstrate the feasibility and efficacy of aPDT against several pathogenic microbes of clinical importance [59,60,61,62,63,64,65]. Within the aPDT field, many photosensitizers and wavelengths have been applied to harness their antimicrobial potential. An important consideration when selecting a PS is the absorption spectrum, which will dictate the appropriate wavelength that should be coupled with the PS [66]. A PS must absorb the light to induce ROS/free radical production in a photodynamic reaction, as described above. The literature has demonstrated the use of different photosensitizers that are amenable to light wavelengths across the electromagnetic spectrum. The combination of UVA with psoralens was mentioned above. However, more relevant example is the combination of UVA with riboflavin, which is clinically used to treat microbial keratitis [67,68]. When UVA (315–400 nm) is combined with riboflavin, it induces ROS that kills microorganisms and aids in the fortification of cornea by increasing its rigidity.

##### Blue Light-Mediated Antimicrobial Photodynamic Therapy

Shifting further to the right of the electromagnetic spectrum, we enter the visible light spectrum at the blue spectral regions (400–470 nm). Blue light, depending on the specific wavelength, may be absorbed by several photosensitizers, such as porphyrin/chlorophyll (405 nm) [69], curcumin (450 nm) [70], or even riboflavin (450–560 nm) [70] to generate ROS that can eliminate several pathogens. Blue light at 400–415 nm in itself has been shown to elicit significant antimicrobial effects without requiring exogenous photosensitizer ([43], see below; Section 2.2) and it has been explored in aPDT research. Studies have used blue light within this narrow spectral region in combination with chlorophyllin [71], chlorin e6 [72], protoporphyrin IX [73], or delta-aminolevulinic acid (5-ALA) [74] to eliminate microbes from food products, in *in vitro* cultures, and *in vivo* in pre-clinical infections and is even applied clinically to manage acne vulgaris [75]. Longer wavelength blue light (450 nm) in combination with curcumin has been used to treat dental infections [76]. Curcumin is a derivative of the plant *Curcuma longa*, a food additive that has numerous health benefits, including antimicrobial, anti-inflammatory, anticancer, and antioxidant properties [76]. 

The potential for curcumin-mediated PDT was evaluated in a clinical study of 13 adult volunteers whose saliva containing several microorganisms were exposed to curcumin, blue light (450 nm), or a combination of the two [77]. A statistically significant reduction of microorganisms (68%) occurred in saliva when compared to curcumin alone (9% increase; *p* < 0.05). While these findings are promising, it is important to appreciate that they were performed on non-infected participants, quantifying microbes within their saliva without active oral infections. Furthermore, the sample size (*N* = 13) was small, which also lowered the potential applicability of the therapy. However, numerous other supportive clinical and non-clinical studies have validated the antimicrobial potential of curcumin-mediated aPDT [78,79,80,81], suggesting that this may be a viable approach to combat infections.

##### Green Light-Mediated Antimicrobial Photodynamic Therapy

Unlike blue light-mediated therapy (or red light; see below), aPDT using green light (495–570 nm) is not applied as frequently and fewer photosensitizers are used in combination with green light. Perhaps the most typical example would be Rose Bengal, a fluorescein derivative that has a peak absorption at 550 nm and has been applied with green light to treat cancer and infectious diseases [82]. Rose Bengal is especially interesting in that it also possesses another function, like the combination of UVA and riboflavin, such that when combined with green light, it can induce collagen cross-linking of the cornea, being a potential treatment for microbial keratitis [83]. Additionally, investigators have proposed and evaluated Rose Bengal plus green light, given its ability to cross-link collagen to facilitate the closure of open wounds via a photochemical bonding [84]. 

Within the infectious diseases’ paradigm, there are a variety of studies that have investigated green light plus Rose Bengal *in vitro* [85,86] and *in vivo* [87], within pre-clinical mouse models, and in human participants [83]. A study shows that Rose Bengal at concentrations as low as 0.03% in combination with low-fluence green light (5.4 J/cm^2^) could significantly reduce the viability of *Staphylococcus aureus* isolates, and at a concentration of 0.1%, this could completely inhibit growth in the dark. Another study showed that concentrations of 50 μM of Rose Bengal in combination with green light (100 J/cm^2^) reduced the viability of *P. aeruginosa* by approximately 4-log_10_ CFU (99.99% reduction), with higher concentrations/light radiant exposures improving the efficacy of aPDT [87]. In their study, however, Rose Bengal plus green light, at the concentrations applied (50–150 μM) or at light radiant exposures, was unable to reduce the viability of methicillin-resistant *Staphylococcus aureus* (MRSA). However, *in vivo* findings were found to be even more modest, with no measurable reduction in viability being achieved. It is important to note that *in vivo* experiments were applied on infected incisional wounds containing a Tegaderm^TM^ bandage, which was found by the authors to increase the bioluminescence signal, which may have potentially obscured the results.

##### Red Light-Mediated Antimicrobial Photodynamic Therapy

Red light (620–750 nm) is perhaps the most frequently used light wavelength applied in aPDT research [88], although it may depend on the country. It is the longest wavelength within the visible spectrum of light, which might make it beneficial in targeting deeper infections (relative to shorter wavelength blue and green light), given that the penetration depth of light is positively correlated with light wavelength [89]. For decades, red light has been coupled with different photosensitizers to treat cancer and infectious diseases [45]. Additionally, red light has been used on its own in low-level light therapy (LLLT), which exploits principles in photobiomodulation to promote wound healing [90], treat dementia [91], or even as a method to overcome traumatic brain injury [92].

When treating infectious diseases, the most applied photosensitizers that are activated by red light are methylene blue [92], new methylene blue [93], porphyrin-based photosensitizers, (such as chlorin e6) [72], and 5-ALA (to stimulate endogenous porphyrin production in cells) [73]. Methylene blue (MB) is perhaps the most well-known and accepted exogenous PS for application in aPDT, given that it is an FDA-approved drug for the treatment of methemoglobinemia [94], and it is used in a variety of medical procedures such as tissue staining [95]. While originally developed as an antimalarial drug [96], its absorption potential at 660 nm makes it an efficient photosensitizer that can generate significant quantities of ^1^O_2_. There are numerous studies that have exploited this to treat a multitude of different pathogenic microbes *in vitro* [97,98,99,100], *in vivo* [101,102,103], and even clinically in human patients [104,105,106]. *In vitro*, at radiant exposures as low as 10 J/cm^2^, coupled with concentrations of MB ≤ 150 uM, MB-PDT has been shown to significantly reduce the viabilities of *Candida* spp. [107], *S. aureus* [108], *P. aeruginosa* [109], *Leishmania* spp. [110], and others, with no significant dark toxicity being observed. In vivo studies, however, have been found to be more modest in their applicability, with numerous studies having exploited MB-PDT to prevent and treat localized infections [52].

In a previous study, MB-PDT was exploited to prevent *A. baumannii* burn infection using the *Galleria melonella* model [111]. In their study, they applied MB (or another PS) to a *G. mellonella A. baumannii* burn infection and measured their survival or melanization rates. They found that over 120 h of observation, larvae treated with MB-PDT had an 80% survival compared with healthy larvae, which had a 90% survival. Larvae that were left untreated had a >50% mortality, demonstrating the potential applicability of MB-PDT in promoting survival. When they looked at melanization, which is an indicator of the relative health status of the larvae, they found that >50% of untreated larvae succumbed to melanization, compared with the MB-PDT group, which had >30% fewer larvae that melanized. Their findings suggested the potential applicability of MB-PDT in controlling localized infections. While these results may not necessarily be perfectly extrapolated towards human participants, numerous clinical studies have supported their applicability [103,104,105,106,112]. For example, MB-PDT was successfully used in two diabetic patients suffering from osteomyelitis of the phalanges of their feet, having not responded well to conventional antibiotic therapy [112]. MB was injected into the affected phalanx and subsequently illuminated internally and externally for approximately 10 min (20–30 J/cm^2^). Over several months, the patients were completely cured. This finding was particularly impactful, as amputation of the digit would have typically been indicated, but MB-PDT effectively removed the necessity for amputation. It is important to note that this study only included two patients. Thus, further work would be necessary. Nevertheless, numerous studies have been conducted or are underway to validate the clinical applicability of MB-PDT [113].

##### Near-Infrared-Mediated Antimicrobial Photodynamic Therapy

There is not a significant amount of literature that has used near-infrared (NIR) radiation in combination with a PS for treating infectious diseases. Like with red light, NIR has been exploited in numerous LLLT studies [114], given that mechanisms within photo-biomodulation mediated by red light apply to NIR, given that broad absorption of cytochrome c oxidase (the chromophore indicated in LLLT) [115]. The most applied photosensitizer with NIR is indocyanine green (ICG), a non-toxic dye used frequently in various medical procedures [116]. It is used frequently for diagnostic purposes, such as determining liver function and cardiac output [116]. In addition, it absorbs wavelengths within the red light and NIR (600–900 nm) spectral regions, which also permits its function as an imaging agent, which is facilitated by the great penetrating capacity of NIR [116]. Furthermore, because it absorbs NIR, it can also function as a photothermal agent and a photosensitizer, generating heat and ROS within target areas [117]. As such, ICG has been experimentally exploited in combination with NIR to combat different infectious agents [118,119] and cancer [117].

For example, due to the deep-penetrating potential of NIR and the excitability of ICG, it was found that the combination could be a potential new approach to combatting lung infections. A study found that ICG under 808 nm irradiation (50 J/cm^2^) could reduce the viability of *S. aureus* by approximately 3 log_10_ CFU *in vitro*, with negligible toxicity being observed against host cells [120]. They also validated the safety and potential for the photodynamic reaction within the lungs via the application of ICG intratracheally, via nebulizer, or other routes, and concluded that not only could photonic absorption be achieved via external illumination (deduced by a photobleaching effect), but there was no evidence of damage to the respiratory tract. The results are very preliminary, although efficacy was validated in a previous study by the same group to have effectively cleared bacteria from the lungs [121]. Although further work is still required, this novel application and potential for NIR to externally reach deeper organs, such as the lungs, opens a window into the potential global applicability of light-based therapeutics to treat localized infections.

### 2.2. Antimicrobial Blue Light

In the section above, we described the photodynamic processes that occur when light across the entire electromagnetic spectrum, including blue light (400–470), is combined with an exogenously applied photosensitizer. Antimicrobial blue light (aBL) represents a relatively new area within the aPDT umbrella because it does not require an exogenous photosensitizer to achieve a photodynamic reaction [43]. To date, multiple studies have sought to understand the intrinsic antimicrobial potential of aBL [122,123,124] and how significant losses in pathogen viability can be achieved. While the mechanism that underpins aBL-mediated microbial killing is not fully understood, it is thought to be driven by endogenous porphyrins that are, apart from a few microorganisms, found ubiquitously. Porphyrin-based compounds are a commonly applied exogenous photosensitizer frequently applied with red light to generate ROS and induce microbicidal effects [125]. Because porphyrins are found endogenously in microbes (and all other eukaryotic cells), the application of 5-ALA (as described above; Section 2.1.2) promotes porphyrin biogenesis within the heme biosynthesis pathway, which may also increase their photoactivity under red light illumination [126].

Porphyrins contain a soret band with a peak absorption at 405 nm, with Q-bands at around 630–660 nm mark [127]. Due to a lower absorption of these Q-band peaks, either porphyrin compounds need to be added exogenously to the microorganism, or porphyrin production needs to be up-regulated to achieve a suitable ‘red light-mediated’ photodynamic reaction [125]. With respect to 405 nm illumination, however, it was found that this is the optimal wavelength that can effectively harness intrinsic antimicrobial effect of the natural and unperturbed porphyrins, likely due to more effective absorption from the porphyrin soret band [43]. Numerous studies have demonstrated the production of many ROS, including hydroxyl radicals, superoxide, and singlet oxygen, all likely contributing to pathogen destruction (Figure 2) [128,129]. In addition, proteins, DNA, lipids, and the cell membrane, have been confirmed to be the antimicrobial targets of aBL [130]. As such, aBL has been studied *in vitro*, including against antibiotic-resistant biofilms [130,131,132], *in vivo* [133,134,135], and in combination with myriad of other conventional and unconventionally used therapeutic agents [135,136,137,138]. Most importantly, aBL, like aPDT, is effective against microbes, irrespective of antimicrobial resistance status [43]. Furthermore, the reduced likelihood of resistance development to aBL [51,139], likely because of the presence of numerous targets, increases its attractiveness as an antimicrobial therapeutic agent. In recent years, it has been discovered that in conjunction with eliminating pathogenic microbes, certain wavelengths within the blue light spectral regions may disarm natural defenses in bacteria, achieved via the photolytic capability of aBL, making it primed for combinatorial approaches [138,140,141,142,143]. For example, in a recent study, aBL (460 nm) was applied to *S. aureus* to destroy the antioxidant carotenoid pigment staphyloxanthin (STX). The authors found that by applying aBL, the STX pigment underwent photolysis, which rendered *S. aureus* unable to tolerate sublethal concentrations of H_2_O_2_ [140].

As mentioned above, aBL has been exploited for *in vivo* pre-clinical use (i.e., animal studies). A recent study from our group found that aBL could effectively rescue mice from a lethal *Vibrio vulnificus* infection [134]. In the study, mice (male or female) that were burned were contaminated with 10^7^ CFU of *V. vulnificus* for either 30 min or 6 h before exposure to aBL. Due to the invasiveness of *V. vulnificus*, it was determined that at 6 h, the mice were considered to have been infected with the organism. It was found that with 120 J/cm^2^ or less, the bioluminescence signal, a quantitative indicator of bacterial burden, could be eliminated from the wounds.

Furthermore, following the maximum radiant exposure of 360 J/cm^2^, reflecting a one-hour exposure, the survival of both male and female mice was significantly improved. For example, in mice infected with *V. vulnificus*, survival was over 80% in female mice, compared with only 30% in those left untreated, indicating a significant improvement in survivability (Figure 2). These findings were particularly impactful, given that *V. vulnificus* infections are associated with high mortality rates (50%) and progress rapidly, suggesting that aBL may be a suitable approach to mitigate mortality associated with *V. vulnificus*.

### 2.3. Ultraviolet Light

Ultraviolet (UV) light (wavelength: 200–400 nm) is a known natural stressor for microorganisms, the use of which in killing pathogens has existed for over a century. This natural antimicrobial activity of UV light is attributed to its potential to cause DNA damage by direct absorption of photons by nucleic acids and the formation of reactive oxygen species (ROS) through a reaction with a chemical substrate (photosensitizer) in the presence of oxygen [143]. Although UV has shown its potential in inhibiting the growth of microorganisms *in vitro* and in situ in infection conditions in clinical cases [144], previous research explored its clinical therapeutic potential through unstandardized protocols [145,146]. Therefore, much information could not be obtained through these clinical reports other than proving the safety and efficacy of these methods. However, recent research has focused on determining the effective and safe dose for using UV light to manage acute infections using standard protocols.

**Figure 3:**
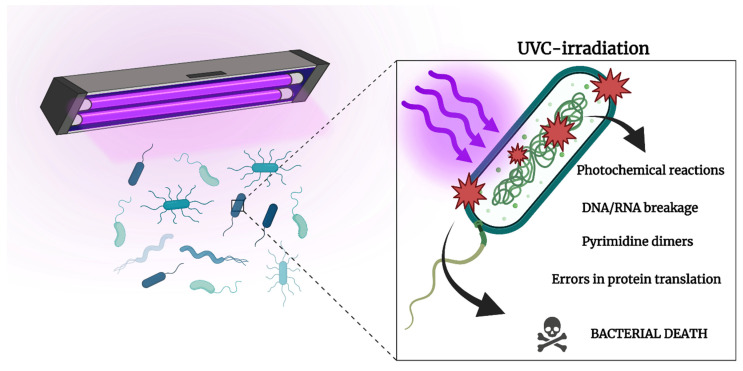
Schematic illustrating the mechanisms of UVC against bacteria. Created on BioRender.com.

Short-wavelength UV (UVC and UVB, wavelengths of 200–280 nm and 280–320 nm, respectively) deactivate microorganisms mainly by affecting DNA integrity (Figure 3). Long-wavelength UV (UVA, wavelength: 320–400 nm) generates ROS and singlet oxygen in the presence of a photosensitizer (e.g., riboflavin), which can damage multiple targets in a cell including proteins and nucleic acids and are lethal to microorganisms [51]. UVA is also intrinsically antimicrobial as it causes DNA damage, but its use in photodynamic reactions using UVA and riboflavin, also called corneal collagen cross-linking (CXL), as described earlier, was originally developed to manage ectatic corneal diseases such as keratoconus. The technology has also effectively managed recalcitrant infectious corneal diseases caused by drug-resistant bacteria, fungi, and *Acanthamoeba* [145,147]. Although CXL is a safe and effective method, it is limited in its application to manage all types of corneal infections daily in an outpatient clinic due to its long and invasive nature that requires corneal de-epithelialization for better penetration of the photosensitizer [147].

Recent investigations have focused on using UVC for multiple acute conditions, such as burn infections and acute corneal infections. Dai T et al. investigated the safety and efficacy of UVC (254 nm) in managing *Candida albicans* infection in mouse third-degree burns [148]. Their *in vitro* studies suggested that UVC could selectively kill the pathogenic *C. albicans* compared with a normal mouse keratinocyte cell line in a light exposure-dependent manner. In the murine model of non-lethal third-degree burn, a single UVC exposure carried out 30 min post-infection caused a 2.16-log_10_-unit reduction in fungal load. UVC was also found to be superior to a topical antifungal drug and was also deemed safe in equivalent doses to the mouse skin. The same group also showed that UVC at 254 nm at a single radiant exposure of 2.59 J/cm^2^ could reduce the bacterial burden in the infected mouse wounds by approximately 10-fold compared to those in untreated mouse wounds. Furthermore, UVC light increased the survival rate of mice infected with *P. aeruginosa* by 58.3% and the wound healing rate in mice infected with *S. aureus* by 31.2%. The authors suggested that UVC light may be used for the prophylaxis of cutaneous wound infections safely and effectively [149]. In a few published reports, Thai et al. showed that UVC could kill *Pseudomonas aeruginosa*, *Staphylococcus aureus*, and methicillin-resistant *Staphylococcus aureus* present in superficial layers of chronic wounds [144]. Pre-clinical research has also confirmed the safety and efficacy of low-intensity UVC in managing corneal infections in mice [150,151]. An exposure of 15 s UVC (1.93 mW/cm^2^), exposed twice daily for two days, effectively managed the *P. aeruginosa* infection [8]. UVC is deemed safe in corneal infections for its poor penetration through the cornea and is likely to be safe to deeper corneal layers [150]. Also, due to the regular desquamation of corneal epithelial cells, it is unlikely that cells retaining defects will pass these defects to subsequent progeny. For the first time, UVC was tested using standard protocols preclinically to manage infections. However, given structural differences in the cornea and other tissues in the body, the doses deemed safe and effective in corneal infections can be different to other body parts and will require further investigations for using this technology to manage all types of superficial infections in the human body.

Similarly to UVA (320–400 nm) and UVC (200–280 nm), UVB (280–320 nm) has also been investigated for its safety and efficacy in managing infections in both clinical and pre-clinical studies. Onigbinde et al. demonstrated that UVB could significantly improve the appearance of decubitus ulcers, reduce the amount of purulent exudate, and fasten skin replacements [151]. However, the literature suggests that UVB can potentially be more harmful than UVA and UVC in terms of carcinogenicity, the fear of which has retarded its investigation into potential infection management.

### 2.4. Can Light Fight against Antimicrobial Resistance?

This manuscript has detailed the mechanisms that drive antimicrobial resistance, alluding to the complexities associated with this concern. While antimicrobial resistance is typically considered an acquired process, we noted that intrinsic and phenotypic resistance processes interfere with appropriate antimicrobial treatment and lead to infection recalcitrance. For example, the intrinsic antibiotic resistance in Gram-negative bacteria limits the application of certain cell wall-targeting antibiotics. Furthermore, the development of biofilms and persister cells tolerate antibiotics due to suppressed metabolic activity, which is vital to facilitate antibiotic function. Therefore, it is reasonable to predict that methods that can universally eliminate microbes, regardless of species or phenotypic status, would be a highly beneficial addition to our antimicrobial armamentarium. Given the nature of light, whether delivered via aPDT, blue light, or UV, it is universal in its applicability towards microbial pathogens (Figure 2 and Figure 3). Given that the photodynamic or nucleic acid degradation processes that drive these light-based approaches are not restricted to any one organism or phenotype (e.g., biofilms/AMR), the identification of infectious etiology or antimicrobial resistance profiles, which would typically be required for antibiotic treatment, becomes less critical. It potentially suggests that ‘light-based’ therapeutic strategies may be applied swiftly and effectively in localized infected regions, thus limiting infection progression and recurrence.

A further benefit of light-based therapeutics is the compatibility with other conventional and non-traditional antimicrobials. One specific example is the ability of certain wavelengths (with or without a PS) that can ‘disarm’ certain intrinsic or acquired resistance mechanisms. aBL, for example, has been shown to ‘permeabilize’ the cell membrane in Gram-negative bacteria, which can promote the uptake of certain antibiotics that would normally be blocked by the outer membrane [152]. Furthermore, aPDT has been shown to inhibit efflux pump expression in bacteria, which might limit the ejection of antibiotics, thus directly overcoming an acquired resistance mechanism [152,153]. β-lactamases [154,155] and virulence factors [156] are other targets that light, under various paradigms, have been shown to destroy or inhibit, further suggesting the potential of light to directly impede AMR.

As with any therapeutic agent, light has several limitations which may hinder its progression towards clinical application. Perhaps the most glaring concern is the penetration of light through the infected regions. Light-based treatments appear convenient when considering infections skin or mouth surface. However, light appears to be out of the therapeutic scope when considering deeper infections, such as necrotizing fasciitis and endocarditis. The use of optical waveguides, however, can increase the feasibility of the method as they permit light to reach any localized region [157]. Nonetheless, these waveguides require significant development, refinement, optimization, and validation for clinical integration.

## 3. Conclusions

It is without question that the variety of mechanisms that drive AMR will eventually leave the population without any useable antimicrobial therapeutics. The intrinsic capacity for microbes to evade antibiotic attack, coupled with phenotypic resistance mechanisms, further complicates the curative potential of antibiotics. In this regard, light appears to have significant potential to combat AMR as an alternative and a method to disarm phenotypes that oppose conventional antimicrobials. However, while there are certain clinical scenarios when light is conventionally applied, a significant amount of work is still required before it can be fully integrated into the clinic. There is a need for further clinical trials to assess the safety and efficacy of different light-based approaches and a requirement to develop innovative waveguides that permit the delivery of light to deeper regions within the human body.

## Data Availability

Not applicable.
